# The unconventional structure of centromeric nucleosomes

**DOI:** 10.1007/s00412-012-0372-y

**Published:** 2012-05-03

**Authors:** Steven Henikoff, Takehito Furuyama

**Affiliations:** Howard Hughes Medical Institute and Basic Sciences Division, Fred Hutchinson Cancer Research Center, 1100 Fairview Avenue North, Seattle, WA 98109-1024 USA

## Abstract

The centromere is a defining feature of the eukaryotic chromosome, required for attachment to spindle microtubules and segregation to the poles at both mitosis and meiosis. The fundamental unit of centromere identity is the centromere-specific nucleosome, in which the centromeric histone 3 (cenH3) variant takes the place of H3. The structure of the cenH3 nucleosome has been the subject of controversy, as mutually exclusive models have been proposed, including conventional and unconventional left-handed octamers (octasomes), hexamers with non-histone protein constituents, and right-handed heterotypic tetramers (hemisomes). Hemisomes have been isolated from native centromeric chromatin, but traditional nucleosome assembly protocols have generally yielded partially unwrapped left-handed octameric nucleosomes. In budding yeast, topology analysis and high-resolution mapping has revealed that a single right-handed cenH3 hemisome occupies the ~80-bp Centromere DNA Element II (CDEII) of each chromosome. Overproduction of cenH3 leads to promiscuous low-level incorporation of octasome-sized particles throughout the yeast genome. We propose that the right-handed cenH3 hemisome is the universal unit of centromeric chromatin, and that the inherent instability of partially unwrapped left-handed cenH3 octamers is an adaptation to prevent formation of neocentromeres on chromosome arms.

## Introduction

The centromere is the most familiar of chromosomal landmarks, having been described by 19th century cell biologists (Flemming 1882). However, the mechanisms that maintain one and only one centromere on a chromosome remain enigmatic. Whereas telomeres and replication origins are maintained by processes that have been described in textbooks for many years (Alberts et al. 1989), just how centromeres are maintained as unique loci on chromosomes continues to be the subject of intense debate (Black and Cleveland 2011). In budding yeast, the genetic basis for centromere identity is well understood, because budding yeast centromeres are genetically specified (Clarke and Carbon 1980). In contrast, the centromeres of other eukaryotes are specified epigenetically, at least to some extent. Even among the yeasts, modes of centromere inheritance vary, from complete genetic specification, as in *Saccharomyces cerevisiae*, to genetic specification without clear sequence specificity, as in *Schizosaccharomyces pombe* (Polizzi and Clarke 1991), to complete epigenetic inheritance, as in *Candida albicans* (Ketel et al. 2009). Multicellular eukaryotes also show a wide spectrum of sequences responsible for centromere specification. For example, arrays of tandem alpha satellite repeat sequences found at native human centromeres can be used to construct artificial centromeres (Harrington et al. 1997), although neocentromeres lacking satellite arrays sometimes appear spontaneously (Marshall et al. 2008). In rice, native centromeres can be composed entirely of satellite sequence arrays or almost entirely lack them (Nagaki et al. 2004). *Drosophila* centromeres are dominated by pentameric and other short repeat arrays, yet no single satellite sequence is found at all centromeres (Sun et al. 1997). *Caenorhabditis* holocentromeres occupy virtually the full length of mitotic chromosomes (Buchwitz et al. 1999) and lack any known sequence determinant (Yuen et al. 2011).

Despite this astonishing variety of sequences found at centromeres, a feature common to virtually all eukaryotes is the presence of a special centromeric nucleosome (Malik and Henikoff 2009). Centromeric nucleosomes are distinguished by the presence of a cenH3 histone (e.g., CENP-A in humans) that takes the place of histone H3. In contrast to canonical H3 and H3.3 histones, which are among the most highly conserved proteins known, cenH3 histones are conspicuously diverged between species and are characterized by distinctive N-terminal tails of variable length and long Loop 1 regions. These major sequence differences between species do not imply functional differences in kinetochore formation, as yeast cenH3 (Cse4) can functionally replace human CENP-A (Wieland et al. 2004). Incorporation of cenH3 nucleosomes is generally thought to determine the identity of epigenetic centromeres. For example, human neocentromeres that form at ectopic sites have no sequence in common and yet are found to be occupied by cenH3 nucleosomes (Warburton 2004). In addition, *Drosophila* cenH3 (CID) is not only absolutely necessary for specifying a kinetochore, but also can be sufficient (Mendiburo et al. 2011). How do cenH3 nucleosomes form the foundation of centromeres and how do they determine centromere identity? To address these questions, we review recent findings on the properties of cenH3 nucleosomes with a view towards reconciling seemingly contradictory observations.

## An altered composition of the budding yeast cenH3 nucleosome?

Until recently, it was widely assumed that centromeric nucleosomes are like conventional nucleosomes in being composed of two copies of each of the four core histones. This assumption seemed justified in that cenH3s contain the same structural elements as canonical H3, despite a higher degree of sequence divergence (Talbert and Henikoff 2010). Furthermore, the structure of the nucleosome containing the H2A.Z variant is very similar to its canonical counterpart despite considerable amino acid sequence divergence. However, a report published in 2007 challenged this assumption with evidence suggesting that cenH3 (Cse4) nucleosomes of budding yeast lack H2A/H2B dimers and instead package DNA with a core particle containing two copies of a non-histone protein, Scm3 (Mizuguchi et al. 2007; Xiao et al. 2011). This evidence was based largely on the ability to form (Cse4/H4/Scm3)_2_ particles in vitro and an evident depletion of H2A/H2B from yeast centromeres.

This model has since been challenged on several grounds. First, Scm3 is a Cse4 histone chaperone (Shivaraju et al. 2011; Stoler et al. 2007), which dissociates from the kinetochore during mitotic exit (Luconi et al. 2011). Similar behavior has been observed for the fission yeast and human orthologs of Scm3 (Dunleavy et al. 2009; Pidoux et al. 2009). Second, the high resolution 3D structure of the Scm3/Cse4/H4 complex indicated that Scm3 blocks histone/DNA contacts (Cho and Harrison 2011). Third, later ChIP mapping showed that Scm3 does not co-map with Cse4 but rather lies immediately adjacent (Camahort et al. 2009). Fourth, Scm3 deletion mutations can be rescued by overproduction of Cse4, indicating that functional Cse4-containing centromeres can form without Scm3 (Camahort et al. 2009). Finally, native ChIP-seq reveals that H2A is as abundant at centromeres as it is genome-wide (Krassovsky et al. 2012), consistent with the documented presence of H2A and H2B in cenH3 arrays of animal centromeres (Blower et al. 2002).

## Evidence for hemisomes at animal centromeres

A second challenge to the assumption of octameric nucleosomes came from a report also published in 2007 that characterized nucleosome particles from native *Drosophila* CID arrays (Dalal et al. 2007). These particles contained all four histones in a heterotypic tetramer, modeled as a half-nucleosome or “hemisome”. This conclusion was based on (1) cross-linking of chromatin and whole nuclei followed by Western blot analysis showing intermediates up to the size of hemisomes, (2) immunoprecipitation of endogenous CID particle arrays and demonstration of equimolar amounts of all four histones, (3) micrococcal nuclease (MNase) digestion showing protection of less DNA by CID particles than by canonical nucleosomes, and (4) atomic force microscopy (AFM) visualization showing an average particle height that is half that of a canonical nucleosome. Electron microscopic visualization also revealed that CID chromatin displays a distinctive beads-on-a-string conformation with long linkers in between nucleosomes and remains decondensed under physiological ionic conditions that cause bulk nucleosomes to condense. Half-height histone cores could be released from CID chromatin and confirmed to contain CID by AFM with recognition imaging (Wang et al. 2008). The lack of DNA in these released particles excludes the suggestion that differences in the DNA wrap can somehow account for the twofold differences in AFM height measurements between CID-containing nucleosomes and those from bulk chromatin (Black and Cleveland 2011).

Hemisomes were later documented in native human CENP-A arrays using AFM with recognition imaging and EM immunolabeling (Dimitriadis et al. 2010). Importantly, this study revealed the presence of a single H2B epitope on the surface of the CID particle that is present in two copies and is internal in the H3 octamer. This study also provided evidence that these arrays indeed derive from centromeres by showing that they are enriched for both alpha satellite and the centromere-specific protein, CENP-C.

In a study published in 2012, sucrose gradient purification was used to isolate octameric CID-containing nucleosomes, and a cysteine cross-linking protocol was used to detect CID-CID interactions, which led the authors to assert that *Drosophila* CID particles are mostly octamers (Zhang et al. 2012). However, the use of digestion down to mononucleosomes meant that centromeric CID arrays, which are uniquely present at centromeres, could not be distinguished from isolated mononucleosomes, which might have been derived by misincorporation of CID into chromosome arms. Moreover, this study drove expression of FLAG-tagged CID with the copia promoter, which has been shown to cause very high level expression (Qin et al. 2010), and as described below, even moderate overexpression of yeast cenH3 leads to misincorporation of octamer-sized particles (Krassovsky et al. 2012). As centromeres correspond to only a tiny percentage of the chromatin landscape, even a low level of misincorporation into chromosome arms might dominate in chromatin preparations but go undetected cytologically, where it could constitute a low background in the presence of intense centromeric spots. Centromeric nucleosomes from both *Drosophila* and yeast are especially sensitive to MNase digestion (Dalal et al. 2007; Krassovsky et al. 2012; Takahashi et al. 1992), and so limit-digestion to mononucleosomes will further favor the recovery of misincorporated particles. The detection of cross-linked CID-CID demonstrated only that octamers existed somewhere in the cells, but whether these were derived from centromeres is unknown. Also, whether or not the cross-linked particles were actually CID-CID, as opposed to CID-H3 or some other cross-linked species was not evident from the evidence presented. As this study did not characterize the DNA associated with the particles that were recovered, the different conclusions between this study and previous ones might be accounted for by a difference between the conformation of isolated CID particles present on chromosome arms and those present in centromeric arrays.

## Right-handed hemisomes at yeast centromeres

**Fig. 1:**
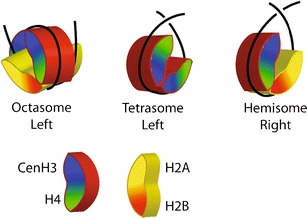
Folding of a left-handed octasome, a (cenH3/H4)_2_ tetrasome, and a right-handed (cenH3/H4/H2B/H2A) hemisome. The octameric histone core is a left-handed spiral of 1–1/2 turns that is held together by highly conserved interaction surfaces between the H2A/H2B of one half nucleosome and the H3/H4 of the other. DNA follows the ramp (*red surface*) created by the spiral to form a left-handed octasome. In contrast, a tetrameric core completes only a three fourth turn, and so DNA wrapping around can cross either above the plane of the particle or below. Tetrasomes are symmetrical and can wrap DNA in either orientation (Hamiche and Richard-Foy 1998), whereas hemisomes are asymmetrical and are found to wrap DNA in a right-handed orientation (Furuyama and Henikoff 2009; Huang et al. 2011)

Rigorous determination of the cenH3 nucleosome conformation is possible using budding yeast, in which centromeres are defined by an ~120-bp sequence. When inserted into a minichromosome, this sequence will mediate accurate segregation to the poles. Previously, yeast minichromosomes were used for in vivo DNA topology analysis to show that Cse4 nucleosomes induce positive supercoils, the opposite of canonical nucleosomes (Furuyama and Henikoff 2009). Loss of a centromere from a minichromosome resulted in a net loss of nearly two positive supercoils, consistent with replacement of a right-handed cenH3 nucleosome with a left-handed H3 nucleosome. The positive supercoiling induced by the deposition of Cse4 nucleosomes was sufficient to conclude that DNA wraps around the particle in a right-handed manner. These results were independently confirmed by the demonstration that positive supercoiling is a feature of the single Cse4 nucleosome at a yeast 2-μm plasmid segregation element (Huang et al. 2011). Right-handed particles are consistent with hemisomes, but not with octasomes, which are held together by highly conserved histone–histone contacts that are lost in particles that wrap DNA in the opposite direction (Fig. 1).

**Fig. 2:**
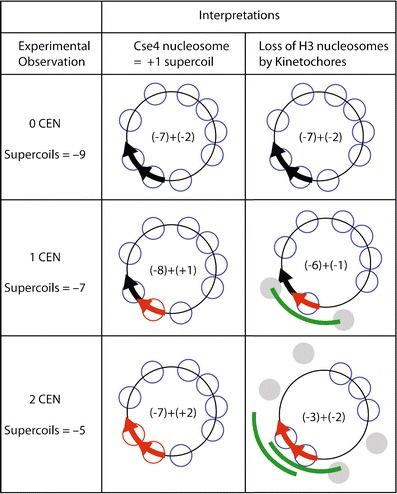
Alternative interpretations of in vivo topology results. Yeast DNA minicircles of identical ~2 kb size were constructed with 0, 1, or 2 functional centromeres (*red arrows*, *black arrows* are non-functional mutated versions) (Furuyama and Henikoff 2009). Superhelical density measurements revealed a gain of ~2 positive supercoils with the gain of one functional centromere and ~4 positive supercoils with the gain of two centromeres. This +2 gain in supercoiling per added centromere is consistent with the replacement of a left-handed H3 nucleosome (−1 supercoil) with a right-handed Cse4 nucleosome (+1 supercoil) (*middle row*). An alternative model is that left-handed octameric Cse4 nucleosomes occupy yeast centromeres (Camahort et al. 2009). To explain the supercoiling data by this model, it has been proposed that the gain of one functional centromere will result in the loss of two left-handed H3 nucleosomes, and the gain of two functional centromeres will involve the loss of four left-handed nucleosomes (Black and Cleveland 2011). Steric hindrance by the kinetochore might plausibly cause exclusion of nucleosomes on either side (*gray*) with gain of one functional centromere (*green line*). However, it does not explain how *two more* nucleosomes are lost with addition of a second centromere in tandem (*bottom row*), since steric hindrance would still only cause exclusion of the two neighboring nucleosomes on either side. Moreover, this interpretation implies that the kinetochore can reach around this small circular chromosome to block nucleosomes more than halfway around

**Fig. 3:**
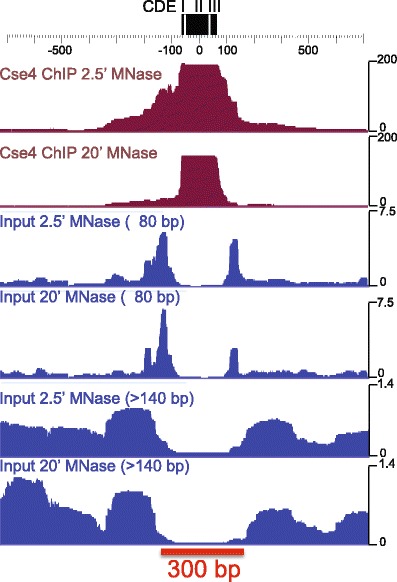
Cse4 maps precisely to the CDE and is immediately flanked by small particles and phased nucleosomes. Cse4 ChIP and input chromatin profiles are based on mapping and stacking of paired-end reads, then calculating normalized counts for each base pair in the interval around the Centromere DNA Element (CDE) of Centromere 3 (Krassovsky et al. 2012). Data for two different MNase digestion time points are displayed. All sizes are shown for the Cse4 ChIP, and small (≤80 bp) and nucleosomal (>140 bp) size fractions are displayed for the soluble (*Input*) chromatin used for the ChIP. Cse4 ChIP corresponds precisely to the CDE, flanked by small particles on both sides, which are themselves flanked by well-phased nucleosomes. Some of these small flanking particles might represent stable protection by Scm3, as they are not detected in the Cse4 ChIP after 20′ MNase digestion (Krassovsky et al. 2012). The *red bar* represents 300 bp, the size of the centromeric gap between centromere-flanking nucleosomes

It has recently been suggested that the topological differences observed between minichromosomes with or without functional centromeres might have resulted from steric hindrance by the kinetochore complex, causing an absence of immediately flanking nucleosomes (Black and Cleveland 2011). Loss of the kinetochore would lead to the appearance of these “missing” H3 nucleosomes on either side and result in a net gain of two negative supercoils, which is topologically equivalent to a net loss of two positive supercoils (Fig. 2, middle row). This is a formal possibility when one considers only minichromosomes with single centromeres, where loss of two flanking H3 nucleosomes would suffice to account for the supercoiling change that was observed. However, this possibility was excluded by the demonstration that loss of one or two centromeres from tandem dicentric chromosomes showed respectively loss of two or four positive supercoils (Furuyama and Henikoff 2009). The steric hindrance model proposed to account for the observed supercoiling changes cannot explain this result, which would imply loss of two flanking H3 nucleosomes from both side of the tandem centromeres or four of the seven H3 nucleosomes on this small circular chromosome (Fig. 2, bottom row). Moreover, the steric hindrance model is directly excluded by the mapping of the two H3 nucleosomes immediately flanking the centromere, which consistently show a gap of ~300 bp between flanking nucleosomes (Fig. 3) (Cole et al. 2011; Gkikopoulos et al. 2011; Krassovsky et al. 2012). With an average nucleosomal repeat length of ~167 bp in *S. cerevisiae* (Lantermann et al. 2010; Tsankov et al. 2010), the replacement of the Cse4 nucleosome by three H3 nucleosomes with loss of the centromere implies that these three H3 nucleosomes fit into an ~300-bp interval where at most only two can fit, a physical impossibility. Although it is formally possible that minichromosomes do not represent the situation in the native chromosomes from which they were derived, decades of studies using yeast minichromosomes indicate that their centromeres function similarly to chromosomal centromeres in numerous different contexts (Rose et al. 1987), and flanking nucleosomes map to identical positions (Bloom et al. 1984). Further evidence for the generality of positive supercoiling by Cse4 nucleosomes comes from the observation that 2-μm segregation elements are positively supercoiled after release of yeast minicircles by targeted recombination from a chromosomal insertion site (Huang et al. 2011). We conclude that the steric hindrance hypothesis cannot account for the changes in topology that led to the conclusion that yeast centromeres are positively supercoiled.

## Single-wrap Cse4 particles occupy yeast centromeres

Positive DNA supercoiling implies a right-handed DNA wrap. Right-handed wrapping is incompatible with stable octasomes, which are held together primarily by the highly conserved interactions between H2A/H2B dimers and the (H3/H4)_2_ central tetramer (Luger et al. 1997). In a right-handed octasome, these surfaces would face away from one another, so would be unable to prevent the particle from springing apart. Although right-handed “reversomes” have been hypothesized to be transiently produced by the positive torsion induced by RNA polymerases during transcription, these particles are estimated to be energetically unfavorable (Lavelle et al. 2009). In contrast, there are no comparable interaction surfaces implicit in the hypothesized structure of a hemisome, and so wrapping might occur in either left- or right-handed configurations, as has been shown to occur for the (H3/H4)_2_ “tetrasome” (Fig. 1) (Hamiche and Richard-Foy 1998).

**Fig. 4:**
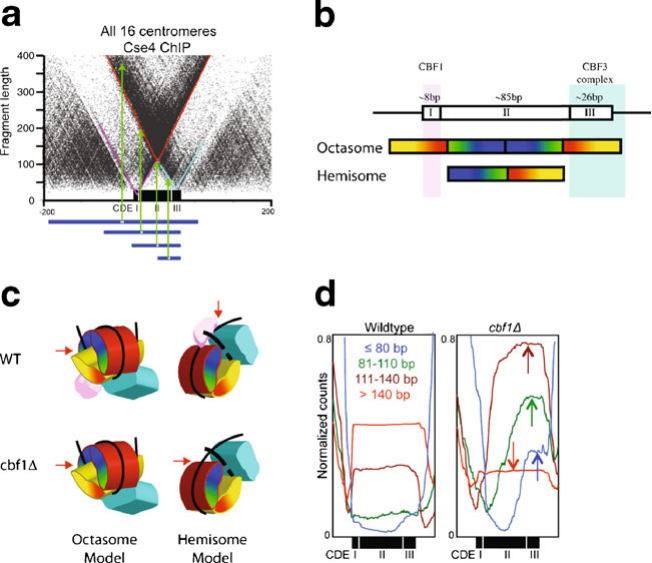
Single base-pair resolution mapping of the tripartite yeast centromere. **a** V-plot representation showing that CDEI, II, and III are each protected from MNase digestion by distinct particles (Krassovsky et al. 2012). Cse4 ChIP was followed by Solexa DNA sequencing library preparation in which particles down to ~25 bp were recovered and paired-end sequenced. A *dotplot* was constructed in which the *x*-axis corresponds to the midpoint and the *y*-axis to the length of each fragment. *Horizontal blue lines* show representative fragments with *green arrows* pointing to dot positions in the V-plot. Note that precise cleavage on one edge of a particle and random cleavage on the other edge generates a diagonal, such that the vertex of the V generated by two edges of a well-positioned particle represents the minimally protected region. The three vertices that are evident in the V-plot (highlighted with *diagonal lines*) correspond to protection of the entire CDE (~125 bp, *red*), protection over CDEI (~15 bp, *magenta*), and protection over CDEIII (~25 bp, *cyan*). **b** Schematic diagram of the CDE showing the extent of protection expected for an octasome and a hemisome which indicates that an octasome must co-inhabit CDEI and CDEIII with Cbf1 and the CBF3 complex. **c** 3D cartoon representation of the folding of an octasome and a hemisome and binding of Cbf1 (*magenta*) and CBF3 (*cyan*). In an octasome, both particles must occupy the surface of the nucleosome, whereas in the hemisome, they would immediately flank the Cse4-containing particle. If Cbf1 is on the surface of an octasome, then its loss should have no effect on MNase protection, whereas if Cbf1 alone is responsible for protection, as predicted by the hemisome model, CDEI protection by the complex would be lost. **d** Deletion of Cbf1 rules out the octamer model. MNase mapping of the yeast genome at single base-pair resolution in wildtype and a *cbf1∆* strain (Kent et al. 2011) shows dramatic changes in the size distribution of paired-end sequenced fragments that protect the centromere (*arrows in right panel*). Two trends are evident: an increase in size of the smaller fragments and a shift to the CDEIII side of the protected peak. This implies that loss of the particle over Cbf1 results in loss of CDEI protection by the centromeric particle and a corresponding reduction in size of the protected region from ~125 to ~100 bp. Such reduced protection is as predicted for a hemisome, but is incompatible with an octasome, where loss of Cbf1 from the surface of the nucleosome would not change the extent of protection. Furthermore, the ~100 bp size of the Cse4 particle in the *cbf1∆* mutant is much less than that of partially unwrapped cenH3 octasomes produced in vitro (Dechassa et al. 2011; Kingston et al. 2011; Tachiwana et al. 2011)

The functional centromere on each of the 16 budding yeast chromosomes is defined by an ~120-bp Centromere DNA Element (CDE) (Densmore et al. 1991). The CDE has a tripartite structure, with an 8-bp CDEI consensus sequence, a 26-bp CDEIII consensus sequence, and an ~90 % A + T 82 ± 4 bp CDEII element in between (Fig. 4a). CDEI is bound by the Cbf1 general DNA-binding protein, and CDEIII by the kinetochore-specific CBF3 complex, both of which sharply bend DNA (Niedenthal et al. 1993; Pietrasanta et al. 1999), leaving only the ~80-bp CDEII element available for full occupation by the Cse4 nucleosome (Meluh et al. 1998) (Fig. 4b). Single cenH3 nucleosomes were first mapped to functional yeast centromeres using chromatin immunoprecipitation (ChIP) followed by indirect labeling with a Southern blot read-out (Furuyama and Biggins 2007). Single base-pair resolution mapping of centromeric chromatin was later obtained using MNase digestion followed by paired-end sequencing of nucleosome-sized particles, which showed that occupancy is precisely delimited to the CDE (Cole et al. 2011). This result is consistent with either tetramers or partially unwrapped octamers. Paired-end sequencing was also applied to Cse4 ChIP and input chromatin, using Solexa library preparation and data display protocols that were developed for single base-pair resolution mapping of both nucleosomal and sub-nucleosomal particles (Krassovsky et al. 2012) (Fig. 4a). The resulting map revealed that the well-established tripartite organization of the ~120 bp CDE sequence precisely corresponds to the tripartite organization of centromeric chromatin. For all 16 yeast centromeres, subnucleosome-sized particles were found to occupy CDEI and CDEIII, with the bulk of Cse4-associated enrichment confined to the ~80-bp CDEII. The three particles over CDEI, II, and III are distinct, because MNase digestion mapping using a “V-plot” representation revealed partial release of both CDEI- and CDEIII-containing particles from association with the Cse4-containing complex (Fig. 4c). Essentially identical V-plot maps were obtained using non-ChIP-based mapping of the insoluble chromatin fraction, which showed an ~100-fold enrichment of kinetochores (Krassovsky et al. 2012). The existence of distinct particles occupying the CDE was confirmed by genetic manipulation, whereby loss of the Cbf1 protein that occupies CDEI caused the expected shift in position and reduction in size of the centromeric protection, which confirmed that CDEI is entirely protected by Cbf1 and is not occupied by part of the Cse4 nucleosome (Fig. 4d). Tripartite centromeric particles were flanked by well-phased nucleosomes on either side, with subnucleosomal particles occupying short linker regions in between (Fig. 3).

This mapping of the Cse4 nucleosome to the ~80-bp CDE of all 16 yeast centromeres implies that there is only a single DNA wrap around the particle, which is inconsistent with an octasome, but it does not distinguish between Cse4/H4/H2B/H2A hemisomes and (Cse4/H4)_2_ tetrasomes. However, ChIP of tagged H2A revealed that the H2A/Cse4 ratio at each of the 16 yeast centromeres is the same as the H2A/H3 ratio throughout the genome (Krassovsky et al. 2012). Taken together with observations of a right-handed DNA wrap and confinement to the ~80-bp CDEII region, the full occupancy of H2A within yeast centromeres indicates that single hemisomes occupy budding yeast centromeres. Taken together with the evidence for hemisomes at animal centromeres described above (Dalal et al. 2007; Dimitriadis et al. 2010), and the observation that budding yeast Cse4 can functionally substitute for CENP-A in human cells (Wieland et al. 2004), it appears that the hemisome is the principal component of centromeres throughout the eukaryotic kingdom.

## Resolving centromere controversies: in vivo observations

The high-resolution mapping of Cse4- and H2A-containing particles to CDEII addresses three controversies surrounding the nature of Cse4 nucleosomes. ChIP had been previously used to argue that cenH3 nucleosomes lack H2A/H2B dimers (Mizuguchi et al. 2007), whereas native ChIP mapping at single base-pair resolution showed that H2A is present at the levels expected for hemisomes (Krassovsky et al. 2012). The earlier report of a deficiency might be attributed to the use of sonication after cross-linking, because any failure to cross-link subunits could result in their loss during sonication, and the degree of loss could differ between H2A/H2B and Cse4/H4. Indeed, analysis of ChIP-chip data from the same group, where they used cross-linking with micrococcal nuclease (MNase) digestion instead of sonication (Luk et al. 2010), revealed the presence of H2A in equal abundance to that observed genome-wide for the six centromeres represented on the microarrays (Krassovsky et al. 2012). Although more recent evidence was suggested to indicate the presence of two Cse4/H4 dimers per nucleosome (Xiao et al. 2011), this conclusion was based on Cse4 ChIP followed by Western blotting analysis of the proteins present in the ChIP material, and any misincorporation of Cse4 outside of centromeres will contribute to the blot signal. Considering that centromeres comprise only ~1/10,000th of the budding yeast genome, a very low level of non-centromeric incorporation can readily account for this result. As described below, Cse4 particles that incorporate on chromosome arms are different from those at the functional centromere and might well include H2A/H2B.

A second controversy concerning the nature of the Cse4 nucleosome was fueled by evidence suggesting the presence of octameric nucleosomes at budding yeast centromeres (Camahort et al. 2009), seemingly at odds with the evidence for positive supercoiling at budding yeast centromeres (Furuyama and Henikoff 2009). The critical experiment that this conclusion depended on was sequential ChIP for two different tags on Cse4, where detection of the both tags implied that the particle had two Cse4 molecules, inconsistent with it being a hemisome (Camahort et al. 2009). However, the use of cross-linking followed by sonication meant that particles around centromeres might have been pulled down as well, and any enrichment of Cse4 in the immediate vicinity of centromeres could have resulted in the ChIP signal reported. Indeed, flanking Cse4 enrichment is conspicuous in high-resolution ChIP maps of native chromatin at centromeres using light MNase digestion (Fig. 3, top track). Even if the double-ChIP signal were derived from centromeric octamers, the data are consistent with a situation in which some centromeres harbor octamers and some hemisomes. The confinement of Cse4 particles to the ~80-bp CDE shown by high-resolution mapping indicates that the large majority of centromeric nucleosomes are single-wrap particles. It is possible that tetramers and octamers exist at centromeres at different points in the cell cycle (Black and Cleveland 2011). For example, octameric assembly intermediates might exist during G1 and split into hemisomes at replication (Dalal and Bui 2010).

A third controversy that high-resolution mapping of Cse4 nucleosomes addressed was a recent assertion by two independent groups that yeast centromeres are not “point” centromeres as has been long assumed (Coffman et al. 2011; Lawrimore et al. 2011). Rather, these authors argued that they are “regional”, consisting of a central Cse4 nucleosome that is flanked by a few randomly incorporated Cse4 nucleosomes nearby. These conclusions were based on quantification of calibrated fluorescence emitted from GFP-tagged kinetochore clusters. A simulation suggested that the original ChIP-based determination of a single Cse4 particle at centromeres (Furuyama and Biggins 2007) was insufficiently sensitive to detect nucleosomes within flanking regions that might lie within a diffraction-limited spot surrounding the kinetochore cluster. However, quantification of the ChIP-seq signal in flanking regions showed that there was at most only ~1–10 % of the Cse4 ChIP density within the estimated span of the kinetochore cluster for all 16 yeast centromeres (Henikoff and Henikoff 2012), excluding this provocative model and confirming the point centromere model for budding yeast. This analysis also provided a caution to the use of external fluorescence standards for quantifying chromatin components labeled in vivo.

## cenH3s form octasomes in vitro

The most persuasive evidence in support of the cenH3 octasome model comes from several nucleosome reconstitution studies, which reveal that octasomes can be readily made from purified components (Camahort et al. 2009; Conde e Silva et al. 2007; Dechassa et al. 2011; Kingston et al. 2011; Mizuguchi et al. 2007; Sekulic et al. 2010; Yoda et al. 2000), including a high-resolution 3D structure of human CENP-A (Tachiwana et al. 2011). This structure revealed that the CENP-A octasome closely superimposes with canonical H3 nucleosomes except at Loop 1, which is 3 aa longer in CENP-A, and the last turn of the N-terminal helix of H3, which is unstructured in human CENP-A. The loss of this helical turn results in an octamer that wraps only 121 bp of DNA, which can account for the smaller size of cenH3 octasomes reconstituted using either human or yeast histones. Partial unwrapping implies that cenH3 nucleosomes are inherently unstable, as previously inferred from in vitro topology studies (Conde e Silva et al. 2007).

The close superimposition of CENP-A and H3 nucleosomes around the dyad axis contradicts a previous 3D structure of the histone-fold domains of (CENP-A/H4)_2_ tetramers, in which the angle between the CENP-A:CENP-A dimers was found to be 9–14° narrower than that for H3:H3 (Sekulic et al. 2010). Such a narrow opening would have resulted in an elongated nucleosome with H2A/H2B dimers making only partial contact. Indeed, an elongated structure with splayed-out dimers was inferred based on small-angle Xray scattering and biophysical measurements for Cse4 octasomes in solution (Dechassa et al. 2011) (although this was not found for CENP-A octasomes (Tachiwana et al. 2011)). Some of these conflicting observations might be reconciled if in solution the lack of a full N-helix allowed unwrapping and subsequent partial collapse of the cenH3:cenH3 4-helix bundle around the dyad axis, whereas the reduced degrees of freedom during the crystallization process might have caused the octasome to “snap” into a more symmetrical conformation in which the electrostatic and hydrophobic interactions are maximized. Although future structural studies will be needed to resolve these differences, what is in common to all of these reports is that the cenH3 octasome is inherently less stable than the H3 nucleosome. Consistent with this conclusion, multiple studies have shown that the highly AT-rich centromeric DNAs that Cse4 nucleosomes occupy in vivo are very poor substrates for octasome assembly (Camahort et al. 2009; Dechassa et al. 2011; Mizuguchi et al. 2007; Xiao et al. 2011). Furthermore, the left-handed Cse4 octasomes that could be obtained using yeast centromeric DNA are unstable, as they were found to spontaneously dissociate after 24 h at 4 °C (Dechassa et al. 2011). Thus, it would appear that yeast centromeres have evolved to exclude Cse4 octasomes, consistent with the fact that runs of As and Ts of the type that are characteristic of CDEII are known to be depleted of conventional nucleosomes and so function as promoters in yeast (Chen et al. 1987; Segal and Widom 2009).

Whereas left-handed octasomes have been reported for human and yeast cenH3s, right-handed particles have been described for *Drosophila* (Furuyama and Henikoff 2009). It is possible that this difference reflects a species-specific difference between the cenH3s and/or partner histones, although it is also possible that differences in assembly conditions used in the different studies are responsible. The conditions used for in vitro assembly are generally non-physiological, and it is possible that the stepwise assembly of left-handed octamers in a strong denaturant followed by dialysis versus 2 M NaCl prior to addition of DNA biased the final product in favor of octamers. Moreover, octamers are expected to be favored over tetramers because they have twice as many electrostatic interactions with DNA, and the use of 2 M NaCl or histone chaperones during assembly are necessary to avoid aggregation during the assembly process. The protocols for nucleosome assembly have been optimized to efficiently produce left-handed H3 octameric nucleosomes (Kingston et al. 2011; Luger et al. 1999), and so it is possible that the production of other forms using these protocols would be disfavored. Alternative methods for nucleosome assembly that are not biased may be needed to resolve this issue.

## Promiscuous incorporation of cenH3s

In the absence of specific DNA targeting sequences, two general models have emerged for the maintenance of epigenetic centromeres. One is that cenH3 nucleosomes are actively recruited by other factors or processes (Foltz et al. 2006), although this begs the question of how the recruiters are themselves recruited if there are no DNA sequence determinants. Nevertheless, it is possible that there are subtle sequence determinants that favor the incorporation of cenH3 nucleosomes.

An alternative model for maintenance of epigenetic centromeres is that cenH3 nucleosomes incorporate promiscuously throughout the genome, but at low levels, and only when they simultaenously occupy long arrays will a kinetochore form (Furuyama et al. 2006). If these occurrences are rare, and the resulting kinetochore inefficient at capturing spindle microtubules, then the devastating consequences of having two functional centromeres would be avoided. Consistent with this interpretation, human neocentromeres are found on chromosomes that have evidently suffered deletions of alpha satellite arrays within the native centromere (Amor et al. 2004; Floridia et al. 2000), as if weakening or loss of a centromere allows for a neocentromere to become established. Likewise, neocentromeres appear spontaneously on *C. albicans* chromosomes when the native centromere is deleted by replacement with a selectable marker (Ketel et al. 2009). Once successful in capturing microtubules and segregating to the poles, these neocentromeres might facilitate further incorporation of cenH3 nucleosomes during subsequent cell cycles and meiotic generations. Neocentromeres tend to form in gene deserts and like native centromeres are associated with heterochromatic marks (Alonso et al. 2007; Lomiento et al. 2008; Marshall et al. 2008), as if genomic regions that are transcriptionally quiescent are favorable for forming epigenetic centromeres. In both maize and *Drosophila*, naturally occurring heterochromatic arrays that are distally located on chromosomes can capture microtubules and orient to the pole, and in the case of *Drosophila*, release of the acentric fragment containing the array results in segregation to the pole (Platero et al. 1999; Yu et al. 1997). These observations suggest that cenH3 nucleosomes can deposit in an untargeted manner, but that they rarely persist long enough or in sufficient local abundance to compete with the native centromere.

Direct evidence for promiscuous cenH3 incorporation has been documented in humans, *Drosophila* and yeast, where overproduction of cenH3 leads to widespread incorporation into chromosome arms (Ahmad and Henikoff 2002; Krassovsky et al. 2012; Van Hooser et al. 2001). Proteolytic mechanisms exist to clear excess cenH3 from chromosome arms in both yeast and *Drosophila*, resulting in normal segregation of chromosomes (Collins et al. 2004; Moreno-Moreno et al. 2006). Mutations in the ubiquitin E3 ligase responsible for proteolytic targeting of yeast Cse4 cause high-level misincorporation and chromosome mis-segregation (Hewawasam et al. 2010; Ranjitkar et al. 2010). In *Drosophila*, high-level cenH3 misincorporation has resulted in the appearance of kinetochore markers at mitosis and chromosome mis-segregation (Heun et al. 2006; Moreno-Moreno et al. 2006), with occasional emergence of competent neocentromeres. These observations suggest that the removal of misincorporated cenH3 from chromosome arms is a normal housekeeping process that can sometimes be overwhelmed, with disastrous consequences.

What is the form of misincorporated cenH3? When budding yeast Cse4 was overproduced ~5-fold and ChIP of MNase-digested material was followed by paired-end sequencing, the size distribution of fragments was dominated by a peak centered over ~135 bp, in contrast to the distribution of input or H2A ChIP material, which peaked at ~155 bp (Krassovsky et al. 2012). This ~20-bp mean fragment size difference corresponds to the size difference between MNase-protected DNA after reconstitution of Cse4 and H3 left-handed octasomes (Kingston et al. 2011), strongly suggesting that even mild overproduction leads to the formation of octameric particles that are incorporated into the genome.

**Fig. 5:**
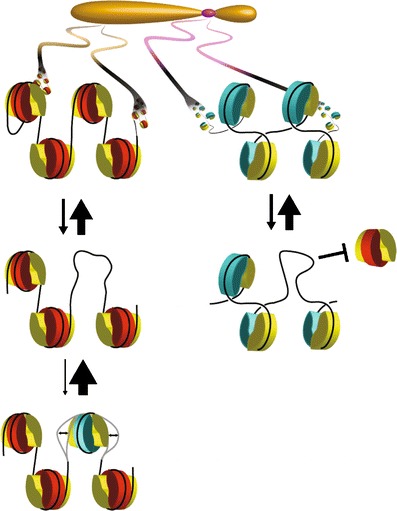
Model for the propagation of cenH3 nucleosomes. Left-handed canonical octamers wrap 147 bp of DNA and dominate on chromosome arms, whereas left-handed cenH3 octamers wrap only 121 bp of DNA and so are inherently unstable (Conde e Silva et al. 2007; Tachiwana et al. 2011). Therefore, the opening of a gap in the nucleosomal landscape by eviction of a pre-existing H3 nucleosome might allow for a cenH3 octamer to form, but it will be readily evicted (*left*). The transience of cenH3 incorporation in euchromatin, where replication-independent nucleosome turnover is frequent, will prevent its accumulation into arrays that are characteristic of native centromeres. In contrast, at heterochromatin-embedded centromeres, the rarity of processes that disrupt nucleosomes, such as transcription, and the presence of heterochromatin- and kinetochore-specific proteins, will inhibit incorporation of H3 nucleosomes (*right*). A stable cenH3 array might occasionally accumulate in a gene desert, forming a weak neocentromere (Platero et al. 1999), which can become the functional centromere if the native centromere is deleted (Amor et al. 2004)

Single base-pair resolution mapping of these octamer-sized particles revealed that they incorporate genome-wide at all nucleosome positions, with peak locations precisely matching peak locations for H2A (Krassovsky et al. 2012). This matching peak distribution between Cse4 and H2A confirmed that cenH3 nucleosomes can incorporate promiscuously at low levels in an untargeted manner. The fact that this strain grew normally implied that such low level genome-wide incorporation of octamers does not result in neocentromere formation. Although peak locations matched between octamer-sized Cse4 and H2A DNA fragments, there was a several-fold bias towards particle incorporation at active promoter regions, which previous work had shown is the site of high levels of nucleosome turnover (da Rosa et al. 2010; Lefrancois et al. 2009). This correspondence between preferential incorporation of octamer-sized Cse4 nucleosomes and preferential eviction of nucleosomes at promoter sites suggests a rationale for the partial unwrapping of Cse4 nucleosomes. Maintenance of the centromere at a unique position on the chromosome requires that any misincorporated cenH3 nucleosomes are evicted before they can organize a kinetochore (Fig. 5). The inherent instability of the cenH3 octasomes implicit in its partial unwrapping might facilitate its removal from “open” chromatin locations, such as promoters, where it preferentially incorporates. Thus, there might be two mechanisms for maintaining chromosome arms relatively free of cenH3 nucleosomes: clashing left-right topological preferences of H3 and cenH3 that preclude formation of stable heterotypic cenH3:H3 octamers and partial unwrapping of homotypic cenH3:cenH3 octamers that results in preferential eviction at sites of misincorporation.

Misincorporation and rapid eviction of cenH3 octamers throughout euchromatin as a conserved housekeeping process might help to explain why some of the same residues at the H3:H3 dimerization interface are also present in cenH3s (Furuyama and Henikoff 2009). Alanine scanning mutagenesis of Cse4 found only six positions where substitutions were lethal, of which five were in the Cse4 dimerization region (Camahort et al. 2009). Of these five, two are bound by the Scm3 chaperone (Cho and Harrison 2011), and Scm3 is essential for kinetochore formation. Another two are involved in interactions within Cse4, and so might be important for protein stability, and not necessarily for dimerization. Indeed, all six of these residues are among the ten that are invariant in both H3s and cenH3s, and so are most easily explained as being essential for protein folding (Henikoff and Furuyama 2010). Nevertheless, to the extent that some of these residues are also essential for cenH3:cenH3 dimerization, this does not necessarily imply a role at the centromere as has been sometimes assumed (Camahort et al. 2009; Zhang et al. 2012), but might reflect the need to avoid forming cenH3 hemisome arrays on chromosome arms.

Whereas the mechanisms that maintain chromosome arms free of cenH3 are expected to be in common between organisms with point centromeres and those with epigenetic centromeres, we expect that there are different mechanisms for the preferential retention of right-handed hemisomes at centromeres themselves. The CBF3 complex that retains the Cse4 nucleosome in *Saccharomyces* and its relatives is absent from other lineages (Malik and Henikoff 2009), which raises the question of how cenH3 nucleosomes are preferentially retained at centromeres while they are being turned over on chromosome arms. As nucleosome turnover that occurs on chromosome arms is an active process that is mediated by RNA polymerases and nucleosome remodelers (Deal et al. 2010; Dion et al. 2007), the absence of such processes at centromeres would result in the preferential retention of cenH3 nucleosomes. Thus, we might think of the compaction and absence of genes in centric heterochromatin as adaptations for preventing turnover that would otherwise evict cenH3 nucleosomes. Consistent with this hypothesis, the appearance of heterochromatic marks at sites of neocentromere formation (Amor et al. 2004) would reflect the formation of compacted structures that prevent turnover events causing replacement of cenH3 nucleosomes.

## Conclusions

Centromeres remain the final frontier of most eukaryotic chromosomes. Despite over a decade having passed since the first draft human genome sequence (Lander et al. 2001), we have yet to see an assembly of human or any other complex centromeric DNA sequences. Moreover, the extraordinary epigenetic basis of centromere inheritance does not conform to any rules that have emerged for other epigenetic processes. We attribute the unique properties of epigenetic centromeres to the unique conformation and topology of the centromeric nucleosome. Although the presence of right-handed tetrameric nucleosomes might seem to be a bizarre solution to the problem of perpetually maintaining a single site on a chromosome available for assembly of a kinetochore, tetrameric nucleosomes are by no means unknown in biology. The genomes of all three ancestral clades of archaea are packaged in tetrameric histone fold proteins (Sandman and Reeve 2006), and recently, the CENP-T-W-S-X complex was shown to consist of a kinetochore-specific heterotypic tetramer of four different histone fold proteins (Nishino et al. 2012). Like cenH3 hemisomes, archaeal nucleosomes wrap DNA in a right-handed manner (Sandman and Reeve 2006), and like cenH3 hemisomes, CENP-T-W-S-X functions in the unique context of the kinetochore, where the task of resisting anaphase pulling forces might require a specialized molecular apparatus (Nishino et al. 2012).

There is as yet no general consensus in the centromere field as to the structure of the cenH3 nucleosome. However, we have argued that the in vivo evidence is consistent in favoring right-handed hemisomes in organisms as diverse as yeast, flies, and humans. This conclusion is especially strong in budding yeast, where in vivo DNA topology analysis, single base-pair resolution mapping, and histone compositional analysis is inconsistent with all other proposed structures. Although left-handed cenH3 octamers have been readily produced in vitro, they are inherently less stable than conventional H3 octamers, and the ability to form metastable structures might allow them to be readily evicted upon mis-incorporation into chromosome arms. The in vivo observation that Cse4 octamer-sized particles can incorporate at all non-centromeric nucleosome positions throughout the genome (Krassovsky et al. 2012) contrasts with in vitro observations that centromeric DNA resists stable wrapping around left-handed Cse4 octamers (Camahort et al. 2009; Dechassa et al. 2011; Mizuguchi et al. 2007; Xiao et al. 2011). Yeast centromeres might have evolved to resist octamer formation, and the confinement of the Cse4 nucleosome to the ~82-bp CDEII region by tightly bound proteins immediately on either side (Krassovsky et al. 2012) would be another means of preventing octamer incorporation at the genetic centromere. A remaining challenge is to find conditions that permit the reconstitution of right-handed hemisomes from purified material on yeast centromeric DNA. Another challenge is to elucidate the pathway whereby right-handed cenH3 hemisomes are assembled in vivo.

Although the ultimate basis for the epigenetic inheritance of centromeres remains a matter of speculation, we are heartened by the tremendous progress made in recent years both in understanding chromatin-based inheritance in many contexts and in elucidating the biochemistry and biophysics of kinetochores. The twin technological revolutions in DNA sequencing and in high-resolution imaging are having important impacts on chromatin and centromere biology, and we expect that a full understanding of centromere function and inheritance is just around the corner.
